# What support is needed to self-manage a rheumatic disorder: a qualitative study

**DOI:** 10.1186/s12891-017-1440-5

**Published:** 2017-02-16

**Authors:** Janet M.J. Been-Dahmen, Margot J. Walter, Jolanda Dwarswaard, Johanna M.W. Hazes, AnneLoes van Staa, Erwin Ista

**Affiliations:** 1 Research Center Innovations in Care, Rotterdam University of Applied Sciences, Rochussenstraat 198, P.O. Box 25035, 3001 HA Rotterdam, The Netherlands; 2 Rheumatology Department, Erasmus MC University Medical Center, P.O. Box 2040, 3000 CA Rotterdam, The Netherlands; 3 Institute of Health Policy & Management, Erasmus University Rotterdam, P.O. Box 1738, 3000 DR Rotterdam, The Netherlands; 4 Intensive Care Unit, Erasmus MC University Medical Center-Sophia Children’s Hospital, P.O. Box 1738, 3000 DR Rotterdam, The Netherlands

**Keywords:** Rheumatic disorder, Outpatient, Self-management support, Qualitative design, Patient perspective

## Abstract

**Background:**

Today, patients are expected to take an active role in the form of self-management. Given the burden of a rheumatic disorder, the patients cannot be expected to self-manage on their own. In order to develop self-management interventions that fit patients’ needs and preferences, it is essential to examine patients’ perspective on how support can be optimized. This study aimed to identify support needs of outpatients with rheumatic disorders and preferences for who should provide self-management support.

**Methods:**

A qualitative study was conducted using focus groups and individual interviews with outpatients with rheumatic disorders treated in a Dutch university hospital. Interview data was analysed with Directed Content Analysis and coded with predetermined codes derived from our model about support needs of chronically ill patients. This model distinguished three types of support: instrumental, psychosocial and relational support.

**Results:**

Fourteen patients participated in two focus group interviews and six were interviewed individually. Most patients preferred an active role in self-management. Nonetheless, they notably needed support in developing skills for self-managing their rheumatic disorder in daily life. The extent of support needs was influenced by disease stage, presence of symptoms and changes in one’s situation. A trusted relationship and partnership were conditional for receiving any kind of professional support. Patients wanted to be seen as experienced experts of living with a rheumatic disorder. Acquiring specific disease-related knowledge, learning how to deal with symptoms and fluctuations, talking about emotional aspects, and discussing daily life issues and disease-related information were identified as important elements of self-management support. It was considered crucial that support be tailored to individual needs and expertise. Professionals and relatives were preferred as support givers. Few patients desired support from fellow patients.

**Conclusion:**

Self-management was primarily seen as patient’s own task. Above all, patients wanted to be seen as the experienced experts. Professionals’ self-management support should be focused on coaching patients in developing problem-solving skills, for which practical tools and training are needed.

## Background

Having a rheumatic disorder requires ongoing psychosocial adjustment and behavioral change to deal with fluctuations, pain, restricted mobility and fatigue in daily life [1, 2]. It may also affect one’s mood, self-esteem, role, relationships, and control perceptions [3]. Today, patients are expected to take an active role [4, 5] in the form of self-management, defined as “managing one or more chronic conditions (e.g. symptoms, treatment, physical and psychosocial consequences, and lifestyle changes) and integrate them in day-to-day life with the aim of achieving optimal quality of life” ([6]: p.547, [7]: p.178). Given the burden of a rheumatic disorder, however, the patients cannot be expected to self-manage on their own; they will need support not only from health care professionals [8] but also from relatives and fellow-patients [4].

Many self-management support (SMS) interventions aimed at patients with a rheumatic disorder are available, including educational programs [9], cognitive behavioral therapy [10, 11] and goal setting interventions [12]. At outpatient clinics, SMS is mostly provided by nurses [13]. There is limited empirical evidence, based on lived experiences [14, 15], on what kind of support outpatients with rheumatic disorders desire. A recent scoping review showed that people with rheumatoid arthritis desire informational, emotional, social and practical support [16]. Another recent qualitative review presented a model of various chronic patients’ support needs distinguishing three types of support: instrumental, psychosocial and relational support [17]. Moreover, professional SMS is often medically oriented, with a tendency to overlook social and psychosocial problems [13, 18]. It should be noted that patients’ support needs are unique and may change over time [17]. Although the self-management tasks patients perceive may be partly disease specific, recent research indicates that self-management support does not necessarily need to be disease-specific since disease type only had a small effect on self-management tasks, and an even smaller effect on support needs [19]. Factors such as flare-up of symptoms, cultural background, gender, and changes in the patient’s personal situation, seem to influence one’s support needs [17].

Professionals could facilitate patients’ self-management by seeing healthcare as a shared responsibility. Patients want to be seen as the daily life experts [20]. Good understanding of patients’ needs could help professionals in designing effective interventions.

We used the model of ‘SMS needs’ [17] to identify what kind of support outpatients with rheumatic disorders need and who they would like to receive support from. This study is part of an intervention mapping process [21] that is expected to lead to the development of a nurse-led self-management intervention that fits patients’ needs and preferences for support.

## Methods

### Design

A cross-sectional qualitative study was applied involving a variety of outpatients with rheumatic disorders and using the directed content analysis.

### Sample and participants

A full sampling strategy was used, inviting Dutch-speaking patients treated at the outpatient clinic of the Rheumatology department of the Erasmus MC, University Medical Center Rotterdam. During seven weeks, three rheumatologists and one nurse practitioner (MW) distributed a flyer with information about the focus groups to eligible patients.

Eligible patients were those diagnosed with rheumatoid arthritis (RA), psoriatic arthritis or ankylosing spondylitis and a minimum age of eighteen years. These patients were recruited because they represent the most common disease of our outpatient clinic. Patients who have been diagnosed recently were excluded. Sixty-three patients were actually invited. Using principles of purposeful sampling [22] in order to create a sample with maximum variation in terms of age, employment, disease type and years of diagnosis, 63 patients were finally invited for group or individual interviews.

### Data collection

Between March 2014 and February 2015, in-depth information was gathered through focus group interviews and face-to-face interviews. Both methods were used because not all patients could attend the focus group sessions. Focus group interviews were considered an appropriate data collection method because participants can be encouraged to discuss and react to others’ remarks. This type of intensive interaction enables a broad exploration of experiences and attitudes, which can enrich data [22–25]. Additionally, individual interviews were held to allow for maximum variation sampling. Individual interviews helped us to gain a deeper understanding of the topics discussed during focus group interviews because participants could explain their view more elaborately. In the analysis, results of both interview types were pooled to develop a comprehensive understanding of patients’ needs and to validate conclusions [22].

The primary researcher, a nurse with basic training in qualitative research methods (JB), conducted the focus group interviews assisted by an independent moderator, a psychologist and psychotherapist who was very experienced with group interaction. This moderator stimulated patients to share their ideas and opinions, but was not involved in data analysis.

These interviews lasted about two hours and were held in a private location outside the hospital. Face-to-face interviews were conducted by JD, an experienced qualitative researcher. These lasted about one hour and were conducted in a private space in the hospital. Leading interview questions are shown in Table 1. Prior to the interview, patients did not receive any information about what kind of support could be provided by whom. This was done in order to encourage them to freely describe their needs for support and preferences for any team member who should provide this support. All interviews were audio-recorded and transcribed verbatim.

**Table 1:** Leading interview questions

- What can you tell me about your life with a rheumatic disorder? - What kind of support do you receive in dealing with your rheumatic disorder? - What kind of support would you need and/or prefer in dealing with your rheumatic disorder? - Who would you preferably like to provide this self-management support?	

### Ethical approval and consent to participate

All invited patients received a flyer and all included participants provided informed consent. Participants were assured of confidentiality and data were processed anonymously by the first researcher. The researchers (JB, JD, AvS, and EI) had no access to patient records, while MW and JH –who were involved in the medical care of some patients- were neither involved in data collection nor had access to non-anonymous data. The study protocol was approved by the Medical Ethical Committee of the Erasmus MC (MEC-2013-350).

### Data analysis

Patients’ support needs were explored through the Directed Content Analysis (DCA) approach, which is appropriate when prior research exists about a phenomenon [26]. Of the two DCA coding strategies, we opted for the one that starts with applying predetermined codes from an existing theoretical framework, in this case the model of ‘SMS needs’ further detailed below [17]. First, the first and second author (JB and MW) read the interview transcripts to gain an overall impression of the contents. Subsequently, they applied predetermined codes based on the different components of the model of SMS needs: (need for support) knowledge – information and instruction, internalizing knowledge, instrumental, adjusting daily life, recognition of emotional aspects, building self-confidence and empowerment, partnership and sympathy. Subthemes of these codes were (support from) professionals, relatives and fellow patients. Factors contributing to the uniqueness of this support were also coded. JB and MW discussed and refined these codes during the coding process. Data considered interesting but which could not be coded with this initial coding scheme were analyzed later *“to determine if they represent a new category or a subcategory of an existing code”* ([26]: p. 1282).

Data saturation was achieved after having analyzed two focus group interviews and four individual interviews when the data became repetitive [22].

#### Theoretical framework: model of SMS needs

**Fig. 1:**
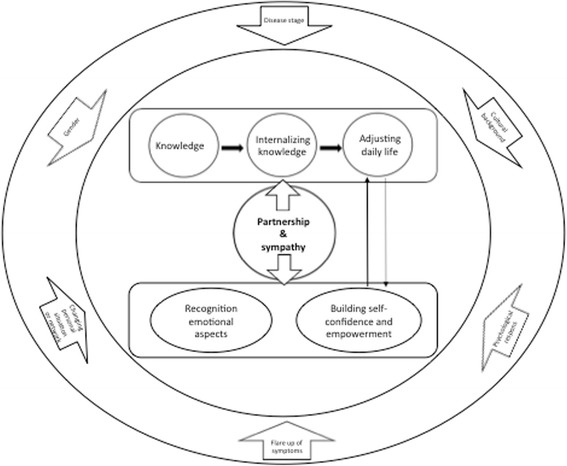
model of SMS Needs [17]

To analyze the data, we used the model of SMS needs (Fig. 1), constructed by Dwarswaard and colleagues (2015). This generic model, developed in a qualitative review of 37 articles, distinguishes three types of support to be provided by professionals, relatives (family and friends) and fellow patients to chronically ill patients: relational, instrumental, and psychosocial [17]. This model of SMS needs will be explained more clearly in the Results section and in Table 2.

**Table 2:** Model of SMS needs [17]

Themes	Subthemes	Quotations to explain the model^a^
**Relational support** refers to supporting aspects of interactions with other persons. This involves two subthemes: partnership, and sympathy.	Partnership	*“It is not possible to hold professionals responsible for everything. It ought to be a matter of co-operation. Every patient should consider what is good for him of her” (II-R5)*
	Sympathy	Patients highly appreciate when their symptoms and side effects are taken seriously: “*Action was taken immediately. In a few days I felt better. I was really accepted” (FG2-R3)*
**Instrumental support** is related to the medical management of a chronic condition, This involves three subthemes: Knowledge–information and instruction, internalizing knowledge, and adjusting daily life.	Knowledge–information and instruction	*“For example, I want information about what can happen if I do not wish to be operated on my hand” (FG2-R4)*
	Internalizing knowledge	Having the opportunity to discuss disease-related information: *“I calm down when a nurse tells me how to interpret side effects I’ve noticed” (II-R4)*
	Adjusting daily life	*“I liked to get advice on how to deal with a rheumatic disorder in daily life. To hear that on the one day you’re capable of house cleaning and the next day you’re not” (FG1-R2)*
**Psychosocial support** pertains to the resources needed to manage the emotional and psychosocial aspects in living with a chronic condition. This involves two subthemes: recognition of emotional aspects of the chronic condition, and building self-confidence and empowerment.	Recognition of emotional aspects of the chronic condition	*“Just ventilating [my emotions or feelings] is enough” (II-R3)*
	Building self-confidence and empowerment	*“For me, it was a psychological transition to inject myself. First, the nurse showed me how to administer this medication. Then she instructed me stepwise. Afterwards I felt confident enough do it myself” (II-R3)*

^a^ Quotations were derived from the focus group (FGI) and individual interviews (II)

### Strategies to establish rigor

Both researcher and method triangulation [22] were used to enhance the validity of the data. All data was collected and analyzed in a team-based fashion. Agreement in coding was reached by consensus between the two coders. To increase the dependability of the research, the design, methods, (preliminary and final) analyses and results were all discussed within the research team. Readers can conclude on the degree of transferability from the provided details of the participants and settings. The description of the methods also contributes to the conformability of this study.

## Results

Forty-three (68%) patients declined to participate, mostly due to logistical difficulties with planning. Eventually, fourteen patients participated in two focus groups interviews (FGI) and six were interviewed individually (II). Sample characteristics are shown in Table 3.

**Table 3:** Sample characteristics

	Face-to-face interviews N (%)	Focus group interviews N (%)	Total N (%)
Gender			
Female	3 (50.0%)	11 (78.6%)	14 (70.0%)
Male	3 (50.0%)	3 (50.0%)	6 (30.0%)
Age			
34–44 years	2 (33.3%)	0 (0.0%)	2 (10.0%))
45–54 years	0 (0.0%)	3 (21.4%)	3 (15.0%)
55–64 years	3 (50.0%)	7 (50.0%)	10 (50.0%)
> 65 years	1 (16.7%)	4 (28.5%)	5 (25.0%)
Marital state			
Cohabiting/married	4 (67.3%)	8 (57.2%)	12 (60.0%)
Widow	0 (0.0%)	1 (7.1%)	1 (5.0%)
Single	2 (33.3%)	5 (35.7%)	7 (35.0%)
Diagnosis			
Rheumatoid arthritis	6 (100%)	10 (71.4%)	16 (80.0%)
Psoriatic arthritis	0 (0.0%)	2 (14.3%)	2 (10.0%)
Ankylosing spondylitis	0 (0.0%)	2 (14.3%)	2 (10.0%)
Years of diagnosis			
< 5 years	0 (0.0%)	1 (7.1%)	1 (5.0%)
5–10 years	2 (33.3%)	7 (50.0%)	9 (45.0%)
> 10 years	4 (66.7%)	6 (42.9%)	10 (50.0%)
Employment			
Yes	4 (66.7%)	2 (14.2%)	5 (25.0%)
No	2 (33.3%)	12 (85.8%)	15 (75.0%)

### Views on self-management

Self-management was primarily seen as one’s own task: *“I want to do it [managing a rheumatic disorder] myself” (II-R1).* Most patients preferred an active role, thinking that others could not manage the rheumatic disorder for them: *“Finally, I’m in charge. I want to experience things myself. Other persons cannot explain everything” (II-R5)*. Ultimately, they themselves have to deal with the disorder: *“in the end no one can really help” (FG1-R1).* Patients wished to “*determine [themselves] what works or does not work […]” (II-R4).* Problems are solved by trial and error*: “Initially, you ask too much of your own body…. But at some point you’ll recognize your limits. To get there, you must be familiar with your own body” (FG2-R2).* Still, actively adapting to the rheumatic disorder can be difficult: e.g. *“Sometimes, I go beyond my physical limits. But eventually, you’ll hit a brick wall” (FG1-R7)*.

### Support needs

Even though self-management was primarily seen as the patient’s responsibility, support from professionals (doctors and nurses), relatives and fellow patients could be accepted. Support might strengthen their empowerment: *“I often have inflammations in my wrist. The pain is terrible. Apart from taking pills, I did not know other solutions. A nurse helped me by sharing the experiences of other patients… At some point I learned to live with it. However, I would like to be guided in managing these challenges in daily life*” (II-R2). Preferences are described below following the ‘SMS needs’ model [17]. Table 2 provides an explanation.

### 1. Relational support

#### Partnership and sympathy

Having a trusting relationship with professionals, relatives and fellow patients was seen as conditional for receiving SMS. Only then, one may comfortably talk about problems at home or work, express one’s own opinion and feel one can rely on the capabilities of the other person. If such relationship is lacking, one may be less open to support: *“I did not want any kind of support from her [a specific professional]… She was not unfriendly, but I did not trust her” (II-R1).* This applies also to relatives and fellow patients: *“First, they [relatives] need to show some genuine interest in me” (FG1-R1)*. Sympathy can affect this level of trust. A sympathetic person was defined as a good listener, someone who is empathic, shows interest and understands.

Continuity of care was important for those who preferred support from professionals. Continuous rotation was seen as counterproductive for building a relationship of trust as becomes clear from a discussion in one of the focus groups: *“At first, I had different doctors. This was very annoying” (FG2-R7). “Yes, that is really annoying” (FG2-R2). “Every time I had to repeat my story. There was a story in the computer,* e.g. *about blood levels. However, this was not my personal story” (FG2-R7)*. Confidence in professionals *“needs to emerge over time” (II-R1)*.

Apart from trust, also partnership with professionals was seen as an important component of SMS: *“It is not possible to hold professionals responsible for everything. It should be a matter of co-operation. Every patient should consider what is good for him of her” (II-R5).* Patients wished to be involved in decision-making and preferred to *“think together about treatment options” (II-R4).* Even though professionals were seen as the medical experts, patients wanted professionals to “*respect the choices” (FG1-R8)* they make. Above all, they wanted to be seen as experienced experts of living with a rheumatic disorder.

### 2. Instrumental support

#### Knowledge – information and instruction

Patients said they needed specific disease-related knowledge (e.g. about diagnosis, symptoms, treatment options, assistive devices, and the necessity of physical exercise). Not everyone needed the same amount and type of information at the same time. Once they had received the diagnosis, patients just wanted information about their rheumatic disorder or how to recognize early symptoms. They did not wish to hear about all possible complications: because, *“I am not ready for it” (FG2-R6).* They were not open to this kind of information until after a certain degree of acceptance has been reached. Some time after diagnosis, patients wanted to receive information related to their personal situation (e.g. about new devices, medication, or symptoms related to complications. Patients’ information needs are also influenced by the disease activity and the symptoms experienced.

In this study, patients preferred a stepwise knowledge provision tailored to personal needs. Failure to provide tailored education carries the risk of patients being *“overwhelmed by all information” (FG2-R6)*. Most patients prefer advice about reliable literature: *“Nowadays, you can find information anywhere. Professionals could help by offering information about reliable sources” (II-R5)*.

In terms of knowledge provision, not much was expected from relatives. However, patients found it important that professionals provide tailored information about the rheumatic disorder to relatives, as lack of knowledge could lead to less optimal support.

#### Internalizing knowledge

Having the opportunity to discuss disease-related information with professionals, relatives, and fellow patients was seen as a way to internalize knowledge. *“I calm down when a nurse tells me how to interpret side effects I’ve noticed” (II-R4)* and *“It helps me to talk with […], someone [a fellow patient] who knows what it means to have a rheumatic disorder” (II-R2).* However, not everyone liked this kind of support from fellow patients: *“I don’t need this [support from fellow patients], because they will constantly talk about their ailment. It gets worse and worse” (FG1-R6).*

#### Adjusting to daily life

Since *“nothing is as difficult as changing your lifestyle” (II-R2),* almost all patients needed support in integrating their rheumatic disorder in daily life. The extent of support need was influenced by the disease stage, the presence of symptoms and changes in one’s situation. Right after diagnosis, more and specific support is needed: *“In the beginning I needed a lot of support. I felt I had my back to the wall. You do not know where it will go” (FG1-R7)*.

Patients highly appreciated professionals who just *“listen and ask how you are doing at home and work” (II-R4).* Besides, professionals could give practical advice about dealing with the disorder: “*peeling potatoes is very hard for me, professionals can advise me on appropriate assistive devices” (II-R4).* Disease fluctuations can be hard to handle. Patients wanted to know how to deal with these.

Some patients needed relatives to monitor their limits: *“Sometimes it is helpful when someone else tells you it is enough” (II-R2).* However, others said: *“I just want to do this all by myself” (II-R1)*. Patients were less ready to accept this kind of monitoring from their children than from their partners. While relatives may provide practical support such as cleaning and cooking, for some patients *“it is difficult to accept help” (II-R5)*.

Most patients said they did not need support from fellow patients. Some acknowledged that *“it is good to know that they [fellow patients] understand how you feel” (FG1-R4)*. Several patients also felt supported by experiential stories in the patient association’s magazine. One patient was active in a social media group because, *“you can ask fellow patients how they are dealing with certain symptoms…. these people face similar problems. A professional does not have this experience” (II-R4).*

### 3. Psychosocial support

#### Recognition of emotional aspects of a rheumatic disorder

Accepting that a rheumatic disorder is a lifelong disease was a deep emotional process for many: *“for me, it felt like an execution” (FG2-R7);* and *“I was really panicking after diagnosis” (FG1-R4).* Mostly it was already helpful when professionals proactively asked and listened: *“just ventilating [my emotions or feelings] is enough” (II-R3).* For some of the patients this was insufficient, however, because they had long-term problems: *“the pain and sadness remain” (FG1-R2).* These patients needed to *“receive guidance” (FG2-R7)* from a specialist e.g. psychologist or social worker, to accept a life with a rheumatic disorder.

Generally, it was easier for patients to discuss emotional aspects when professionals proactively asked about these. Not all patients had the courage to discuss these kinds of problems, sometimes because they *“do not want to be perceived as a bore” (FG1-R1).* Patients preferred to discuss emotional issues with a nurse, because nurses tended to be *“able to create a moment to listen” (FG1-R6).*

Most patients just wanted a listening ear from relatives, but some pointed out that relatives did not always recognize their emotional issues. Not all relatives were able to *“imagine what it is to be a chronic patient with daily pain” (II-R4).* As a result, not all patients received the support they needed. Compared to children and friends, partners seemed more capable in recognizing such emotional issues.

Fellow patients could be of help when they have the same experiences: *“I want to talk with someone who is experiencing the same” (II-R4).* However, patients were not interested in meeting fellow patients in a group session organized by the hospital. Some patients preferred to meet them informally.

#### Building self-confidence and empowerment

Although described implicitly, encouragement and reassurance supported the building self-confidence and empowerment: *“For me, it was a psychological transition to inject myself. First, the nurse showed me how to administer this medication. Then she instructed me stepwise. Afterwards I felt confident enough do it myself” (II-R3).* Positive reinforcement seems to help patients to solve problems or change behavior. For example, when a physician told a patient “*that she would be able to exercise” (FG1-R7)* and that it should help her, she felt confident to exercise more often so that her body became more flexible. It could also be helpful to see other patients exercising*.* On the other hand, some thought it would be confrontational to see the consequences of rheumatism in others.

## Discussion

In this qualitative study we explored the support needs of people living with rheumatic disorders. The analysis learned that they saw self-management primarily as a task for themselves but nevertheless appreciated support to help them achieve this. Most of the interviewed outpatients preferred support from professionals and relatives; only few appreciated psychosocial support from fellow patients.

Although the concept of self-management assumes an active role for patients in managing and integrating a chronic condition(s) in daily life [6, 7], it was striking to find that this concept seems to fit so well to outpatients with a rheumatic disorder. However, even when patients appear to be autonomous self-managers their need for support should not be underestimated. It is not reasonable to expect patients to manage a rheumatic disorder on their own [27, 28]. All patients need encouragement [29] to develop enough self-confidence to manage a disorder. Bandura found self-efficacy to be an accurate predictor of patients’ fulfillment in managing a disorder [30, 31]. The core element of professionals’ support should therefore be coaching patients to develop problem-solving skills. It should be remembered, however, that not everyone believes in their capacity of self-managing. Patient with less confidence need more encouragement and recommendations from others e.g. professionals and relatives [32].

We found that learning how to deal with symptoms and fluctuations, talking about emotional aspects, and discussing daily life issues e.g. work and household were important aspects. These are all important aspects of the broad definition about self-management [6, 7], indicating that patients with rheumatic disorders are challenged to deal with the medical, emotional and social issues of their disorder in daily life [8]. These aspects also came to the fore in two reviews [16, 17]. One of these reviews shows that patient-related factors influence support needs [17]. In this qualitative study we did not found any difference for gender, age and work status. Moreover, the time since diagnosis and course of the rheumatic disorder affected support needs. Patients who perceive their disease activity as unstable and who experienced more disease activity, in line with previous research [33]. It would be worthwhile to study how SMS could be tailored to individual needs and expertise [34].

The interviewees in this study saw partnership and a trusted relationship as conditional for receiving SMS. Continuity of care and professionals taking the problems seriously could help build a trusted relationship. A good professional-patient relationship is therefore the cornerstone of care, especially in view of achieving behavioral change [35, 36]. Partnership is generally recognized as an important part of SMS [8, 37, 38]. However, it can be difficult for professionals to achieve collaborative partnership [39–41] as they may be inclined to play the expert role [18]. Patients in this study appreciated support from nurses and doctors alike. Usually, nurses took more time to discuss emotional and social aspects.

Partnership and a relation of trust were not only conditional for support from professionals, but also from relatives and fellow patients. Relatives were especially prized for their emotional and practical support. Fellow patients can help by sharing their lived experiences. However, not all patients appreciate this kind of support, unless this can help in managing a chronic condition well [4]. Modeling, observing others in performing new behavior patterns successfully, can serve as a guide for translating behavioral conceptions to appreciate actions [27].

Operationalizing SMS may not be easy for professionals [13]. They tend to resort to traditional (standardized) patient education [18], instead of providing the recommended tailored patient education [42]. Moreover, interventions that solely provide education have been found least successful [43, 44]. Interventions focusing on patients’ intrinsic processes seem to be most successful [45]. Focusing on more internal perceived locus of control is important for persistence and performance of new behavior [46]. Still, professionals lack skills to facilitate psychosocial challenges in self-management [38]. Additional training could help professionals to incorporate coaching into their repertoire of SMS interventions.

The model of ‘SMS needs’ (Fig. 1) [17] we employed was helpful in that we benefitted from previous descriptions and could create a deeper understanding of the support needs of people with a rheumatic disorder. On the other hand, the DCA approach carries the risk of fitting data to the predetermined coding scheme. Relevant data can be missed when applying this highly-structured method. To minimize this risk, we also applied inductive coding if data could not be categorized. This enabled us to unravel the importance of the ‘self’ in self-management for patients with rheumatic disorders. Lastly, collecting data from not only focus group interviews but also face-to-face interviews was very useful. Individually interviewed participants in elaborated more on their experiences, which helped to create a comprehensive understanding of patients’ needs. However, findings from the two interview types did not differ essentially.

A possible limitation of this study is that mostly elderly, retired patients with RA participated in the focus groups. It was difficult to recruit younger persons for the focus groups. Still, given that the prevalence of RA is much higher than the prevalence of psoriatic arthritis and ankylosing spondylitis, that RA occurs at older age, and that most of the RA patients were women, the composition of our sample seems to correspond to the normal distribution in the general population [47]. However, to minimize the risk of selection bias, we purposefully searched for younger or employed patients for the individual interviews.

In this study, we decided to exclude patients who have been diagnosed recently and to ask patients in retrospect what their supports needs were at the time. We did not specifically study the support needs of recently diagnosed patients. Furthermore, all data was collected in one hospital in the Netherlands and the findings may therefore not be representative for patients in others countries. Hence, we recommend to study whether of outpatients in other countries may perhaps have other SMS needs.

## Conclusion

Self-management was primarily seen as one’s own task, but patients still appreciated support to help achieve this. Above all, they wanted to be seen as experienced experts of living with a rheumatic disorder. Preferred support givers were professionals and relatives. Professionals’ self-management support should be focused on coaching patients in developing problem-solving skills for managing the medical, emotional and social challenges experienced in dealing with a rheumatic disorder in daily life. Practical tools and training are needed to operationalize coaching as a part of professional self-management support in working routines.

## Abbreviations

DCA: Directed Content Analysis
FGI: Focus group interview
II: Individually interview
RA: Rheumatoid arthritis
SMS: Self-management support
